# Pediatrician involvement in communicating positive newborn screening results for Krabbe disease: barriers, facilitators, and ideas for interventions

**DOI:** 10.1186/s13023-026-04459-3

**Published:** 2026-06-17

**Authors:** Laura Kirkpatrick, Erin Friel, Gysella Muniz, Judy C. Chang, Deepa S. Rajan

**Affiliations:** 1 Departments of Pediatrics and Neurology, University of Pittsburgh, UPMC Children’s Hospital of Pittsburgh, 4401 Penn Avenue, Pittsburgh, PA 15224 USA; 2 Department of Pediatrics, University of Pittsburgh, Pittsburgh, USA; 3 UPMC Children’s Hospital of Pittsburgh and UPMC Magee Women’s Hospital, Pittsburgh, USA; 4 Departments of Obstetrics, Gynecology and Reproductive Sciences, Department of Medicine, and the Clinical and Translational Science Institute, University of Pittsburgh, Pittsburgh, USA; 5 Department of Pediatrics, University of Pittsburgh and UPMC Children’s Hospital of Pittsburgh, Pittsburgh, USA

**Keywords:** Krabbe disease, Implementation science, Newborn screen, Pediatrician, Communication, Qualitative

## Abstract

**Background:**

In 2024, the U.S. Department of Health and Human Services added infantile Krabbe disease to the Recommended Uniform Screening Panel for Newborn Screening (NBS). Families have previously expressed wanting their established general outpatient pediatrics clinicians involved in sharing NBS results. In this study, we aimed to understand pediatrics clinicians’ perspectives on barriers, facilitators, and ideas for interventions regarding their involvement in sharing positive NBS results for Krabbe disease.

**Methods:**

We conducted individual semi-structured interviews with clinicians (physicians and advanced practice providers) in general outpatient pediatrics in Western Pennsylvania. Interviews were audio-recorded and transcribed. Two coders performed content analysis, coding transcripts, and identifying themes with representative quotations. Themes were mapped to the Consolidated Framework for Implementation Research.

**Results:**

Twenty-five clinicians participated. Barriers, facilitators, and ideas for interventions spanned the individual level, inner setting, and outer setting of the Consolidated Framework for Implementation Research. Barriers included: limited clinician knowledge of Krabbe disease, time in the clinical schedule, and accessibility of specialists. Facilitators included: prior clinical training in communicating serious diagnoses, protected time for Continuing Medical Education activities, and trustworthy online medical resources. Ideas for interventions included: fact sheets/scripts/algorithms for clinicians; integration of clinician education with NBS result notifications; and improvements in care coordination with specialists.

**Conclusions:**

While primary care pediatrics noted lack of time, knowledge, and access to specialists as barriers to sharing Krabbe disease newborn screening with families, they highlighted that communication tools/tips, easy training or access to disease information, and assistance coordinating specialty care would facilitate their involvement.

## Introduction

Krabbe disease is an autosomal recessive condition caused by toxic accumulation of psychosine due to deficiency of the lysosomal enzyme galactosylceramidase [1–3]. Krabbe disease is rare, affecting 1 in 100,000 live births in the United States [1–3]. The classical infantile-onset Krabbe disease is a rapidly progressive leukodystrophy, which presents around 6 months of life with irritability and developmental arrest or regression then ultimately rapidly progresses to worsening disability and death often by 2 years of life if untreated [1–3]. Allogeneic hematopoietic stem cell transplantation (HSCT) in presymptomatic infants has been shown to slow progression of the disease [1–3], but does not halt the peripheral neuropathy which continues to progress [1–3]. While HSCT is not curative it is currently the only available disease modifying treatment option. Emerging investigational approaches include gene therapies, substrate reduction therapy, and CNS penetrant enzyme replacement therapies, that are currently in preclinical stages of development.

To enable prompt identification of pre-symptomatic children and timely arrangements for HSCT, several U.S. states expanded routine Newborn Screening (NBS) to include testing for Krabbe disease, including Pennsylvania in 2021 [1–4]. Initially, due to concerns of high false positive rates, uncertain prognosis and insufficient evidence of presymptomatic treatment benefit, the DHHS Advisory committee rejected addition of Krabbe to the RUSP. But since then with the addition of psychosine to the NBS as second tier testing and subsequent improvement in specificity, the U.S. Department of Health and Human Services added infantile Krabbe disease to the Recommended Uniform Screening Panel, a list of conditions recommended for inclusion in state NBS programs in 2024 [5]. Currently, 19 states include Krabbe on their Newborn screening panel. Outside the United States, the population level screening of Krabbe has not been broadly implemented, possibly due to the limited availability of HSCT options and ongoing uncertainty of natural history of those treated in early or pre symptomatic stages.

Inclusion of Krabbe disease on the NBS necessitates ensuring that positive results are communicated to families in a timely, informative, and sensitive manner with a clear plan for next steps in their child’s care. Prior qualitative work with families has indicated that they prefer their established pediatrician be responsible for sharing information with them about NBS results, accompanied by rapid referral to a specialist [6–10]. However, little is known about the readiness of U.S. outpatient general pediatricians to communicate and discuss positive NBS results particularly for rare neurometabolic conditions such as Krabbe disease. To understand how to best integrate general pediatricians into this care process, we conducted semi-structured interviews with clinicians (physicians or advanced practice providers (APPs)) working in outpatient general pediatrics in Western Pennsylvania, a region where Krabbe disease is included on the NBS. We inquired about barriers, facilitators, and ideas for interventions regarding general pediatrician optimal involvement in counseling and communication with families about positive NBS results for Krabbe disease.

## Methods

### Recruitment

We recruited individuals for interviews through emails to practicing clinicians in three outpatient pediatric health systems in Western Pennsylvania. Attending physicians and APPs in general outpatient pediatrics practicing in Western Pennsylvania were eligible. We used purposive sampling to ensure diversity in provider type (physician or APP), years in practice, practice type (academic or community/private), and practice setting (urban, suburban, or rural). Participants received a $50 gift card for their time. We planned to enroll participants until reaching thematic saturation (i.e. when no new themes emerge from further qualitative data collection).

### Interview guide development

We developed a novel interview guide concerning general outpatient pediatricians’ perceived barriers, facilitators, and ideas for interventions for their involvement in positive NBS result disclosure for Krabbe disease. Questions and prompts were grounded in the Consolidated Framework for Implementation Research (CFIR) [11]. CFIR is a framework to help guide researchers in a systematic evaluation of barriers and facilitators to clinical implementation of an evidence-based practice [11]. The interview guide was reviewed by an expert in neurogenetic diseases including Krabbe disease (D.R.) and a general pediatrician with expertise in newborn care (G.M.).

### Interviews

One team member (E.F.) conducted all the individual semi-structured interviews. She is a female research coordinator working with the P.I. (L.K.) with extensive prior training and experience in qualitative research. She had no prior relationship with any interviewees. Participants were aware that she is a research coordinator working with the P.I. but no other information about E.F. was provided to them. Participants were aware that they were engaging in semi-structured interviews about general outpatient pediatricians’ perceptions of barriers and facilitators to their involvement in sharing positive NBS for Krabbe disease in Pennsylvania. Interviews occurred either by telephone or by Zoom depending on participant preference. Participants could engage in interviews from any physical location of their choosing. All interviews were recorded and transcribed verbatim.

### Analysis

The study team developed a preliminary codebook based on anticipated categories of feedback and domains of the Consolidated Framework for Implementation Research [11]. Using a qualitative content analysis approach [12], two coders (L.K. and E.F.) applied the codebook to all transcripts deductively and inductively added new codes as needed when new topics and concepts were noted. The two coders met periodically throughout the coding process to review and reconcile their coding and agree upon creation of any new codes. A senior co-investigator (J.C.) was available to adjudicate any discrepancies of interpretation. The two coders reviewed the coding data and ascertained themes related to barriers, facilitators, and ideas for interventions regarding integration of outpatient general pediatricians into optimal NBS result disclosure for Krabbe disease in Pennsylvania.

### Ethical approval

The University of Pittsburgh Institutional Review Board determined this study to be exempt from formal review.

### Data availability and materials

Deidentified data is available to any investigator upon request.

## Results

25 providers engaged in semi-structured interviews. The sample included 19 physicians and 6 APPs with a median of 9 years in practice (interquartile range 5–20). All clinicians had disclosed NBS results for at least one condition, but none had disclosed positive results for Krabbe disease specifically. Ten clinicians had prior experience treating a patient with Krabbe disease. We achieved thematic saturation after the 22nd interview, then performed three additional interviews to confirm saturation. Detailed demographics area available in Table 1.

**Table 1:** Participant demographics

Years in Practice (median (interquartile range))	9 years (5–10 years), range 1–39 years
Provider Type (n (%)) APP Physician	6 (24%) 19 (76%)
Sex (n (%)) Female Male	19 (76%) 6 (24%)
Race (n (%)) Asian Black White	4 (16%) 1 (4%) 20 (80%)
Ethnicity (n (%)) Non-Hispanic	25 (100%)
Practice Type (n (%)) Academic Combined Private and Academic Federally Qualified Health Center Private Practice	6 (24%) 1 (4%) 2 (8%) 16 (64%)
Practice Environment (n (%)) Rural Suburban Combined Suburban and Urban Urban	2 (8%) 11 (44%) 1 (4%) 11 (44%)

### Themes

Several barriers, facilitators, and ideas for intervention emerged regarding integration of pediatricians in optimal positive NBS result disclosure for Krabbe disease in Pennsylvania related to the different levels of the Consolidated Framework for Implementation Research [11]. These included the individual level (i.e. characteristics of the clinician), inner setting (i.e. clinic environment), and outer setting (i.e. broader context such as the health system, regional, state or national environment). Key themes related to barriers, facilitators, and ideas for intervention with illustrative quotations are described in the text below as well as in Tables 2, 3 and 4.

**Table 2:** Barriers to optimal disclosure of positive newborn screen results for Krabbe disease in Pennsylvania

CFIR 2.0 Domain	Representative Quotation
Individual-Level	
Limited knowledge of Krabbe Disease	“I do think some of the metabolic screens that we’re not as familiar with there is still some confusion on. Like, ‘Who do I follow up with this on?‘” -- Participant #22 “The biggest [barrier[ would probably just be, you know, the knowledge basis of the disease and maybe also just lack of experience” - Participant #20 “Anything you do, you have repetition on, you are going to be awesome at. And so Krabbe disease is not, a disease that I can give a lecture on without any notes, right? I need to…refresh my memory.” - Participant #17 “ I think [a major barrier] would just be if the provider feels like he or she or they don’t have enough knowledge on it to be able to appropriately sort of inform and counsel and provide next steps.” - Participant #10
Limited interest in learning about rare diseases	“[I’m] maybe moderately interested [in learning about Krabbe disease] just because it’s not something I see all the time, that I may not be super excited to see it.” - Participant #20 “Ten being super motivated and one being not all, I would say probably like a five [in motivation to learn about Krabbe disease].” - Participant #19
Limited comfort in disclosing serious news	“I think it’s always difficult to disclose …, something that would strike so fast and so soon… [it would] just be uncomfortable for anybody” - Participant #25 “ Well, I mean, specifically for Krabbe that there’s no cure. That would definitely be challenging to address or discuss.” - Participant #19 “I think the other thing that is always hard for any person to talk about is…it’s a fatal disease. How much longer do I have with my child? What’s my child’s quality of life gonna be? All those kinds of questions, I think, sometimes make us feel uncomfortable because we’re so used to dealing with well children.” - Participant #9
Inner Setting	
Limited time in the clinical schedule	“I mean, honestly, time. [is a barrier]…I feel like there is a big push to see more patients in less time.” - Participant #22 “ Doing your job well typically always takes a lot more time. And time is precious, and you don’t have tons of it. - Participant #14 “I think definitely challenging would be um, we only get like 30-minute slots, and so this might take a little more time—um, with the family. So, I think that would be challenging, having that protected time to, you know, be there for them, answer all the questions, things like that.” -Participant #3
Lack of a pre-existing relationship between the clinician and the family	“It’s patients that kind of show up and you don’t have a good and a preexisting long-term relation and-and trust with them and breaking news like that, can be much-much more difficult. And it’s either you get to—but it’s time to get—we get know each other very well now, or it’s that they lose trust in you immediately, and they may shut down and that’s really kind of difficult to predict.” - Participant #13 “[It feels] uncomfortable delivering that news, especially if you haven’t met the family or meeting for the first time or met them just once if that’s—it’s their first kid you’re seeing.” - Participant #5
Limited family knowledge of Krabbe disease	“I don’t know that they would really understand, um, the seriousness of that disease” - Participant #16
Difficulties connecting with families	“Then barriers to like, getting in touch with the family in an appropriate manner if that was a factor.” - Participant #24
Outer Setting	
Release of results to families before clinicians can disclose the results first	“I think one of the challenges with newborn screens is that if the information is relayed to the parents ahead of the provider being able to tell the parents- that’s the first problem because I think it’s best that the provider should be able to sort of explain what the findings mean.” - Participant #10 “I actually find it on many levels wrong for the [health department] to send [the family] a letter before notifying me first.” - Participant #6 “[The family] may read, the worst-case scenarios, an it’s hard for them necessarily to put their own child in context” - Participant #15
Inefficient transmission of NBS results to clinicians	“I think there are rare occasions where a newborn screening test is done, but the results don’t come to us. And we see a one-month-old and we look back to see what the newborn screen was. And normally, the newborn screening result comes in. It’s seven or 10 days old at-at most. But I’ve had patients who were a month old, and I don’t have a record of the result, and I don’t know who it went to, and I don’t know who reviewed it.” - Participant #4 “Sometimes in the office we have issues with the screens coming back to us and sometimes depending upon where the patient was seen as a newborn and how they were registered, like, their name and date of birth, at birth, as opposed to when we see them in the office, sometimes there’s a lag—- so making sure that those results are readily available at the appropriate time so we can address them as soon as we can.” - Participant #8
Inaccessibility of specialists with expertise in Krabbe disease	“Lack of being able to communicate with the specialist directly if you had a question [is a barrier].” - Participant #24

**Table 3:** Facilitators or ideas for interventions to promote optimal disclosure of positive newborn screen results for Krabbe disease in Pennsylvania

CFIR 2.0 Domain	Representative Quotation
Individual-Level: Provider	
Training and experience in communicating serious news	“[The training] helps them feel a little bit more prepared, perhaps, when that first time that they have to disclose something uncomfortable, um, to a family, about a serious illness in a child. It gets easier over time.“- Participant #9 “I think it comes down to training and making sure that pediatricians in general are well versed andcomfortable with disclosing bad news. It’s not always sensitive to say it’s bad news, but in the end, that’s what it is.” - Participant #13
Strong sense of responsibility to patients and families	“I do think it is part of my job” - Participant #25 “It’s not like newborn screening system reports it to the subspecialist, so the pediatrician’s gonna be the one to have to name it, say it, and send the family, um, to someone else.” - Participant #9
Intellectual curiosity and motivation to learn	“I’m super motivated [to learn]. I think it’d be great.” - Participant #16 “I would always be open to-to learning more about [Krabbe disease]. I hope I never have a patient with it, but knowing that it’s a possibility that I would and would have to the person who’s kind of disclosing that… I absolutely would, you know, be motivated to know more about it.” - Participant #25
Inner Setting	
Protected time for Continuing Medical Education	“You know, I think it’s one of those things that a lot of the diseases that we test for are obviously rare. So we just don’t—you know, over time… you can forget what you learned at school about those rare diseases. So I think periodic updates would be super helpful. Um, because honestly, I can tell you, if that came across my desk today, I would freak out a little.” - Participant #18 “My organization does a ‘lunch and learn’ presentation for Continuing Medical Education about newborn screens and abnormal newborn screens. They’ll send a PowerPoint presentation recording of the lecture even if I’m not able to attend, and I have that in my email to search so that I could pull that up.” - Participant #4 “So for our clinic in specific, we have a meeting every Thursday, and sometimes we also have people come to present like educational topics at that meeting. So, that could be an option to get it out there to everybody ‘cause that’s like protected time for our learning.” - Participant #3
Outer Setting	
Accessibility of specialists	“Probably [I would talk to] genetics -- like discuss how they would treat or manage the patient so I can give them a little bit more expectation.” - Participant #19 “I’m lucky because I’ve got a top 10, I think, children’s hospital [laughter] in < City> that’s nearby that I can just consult. I can call the consult.” - Participant #13
Trustworthy online medical resources	“[I would prepare for the appointment by] going through a generally available resource like UpToDate” - Participant #4 “I get UpToDate, and I think the AAP has good information out there” - Participant #22

**Table 4:** Ideas for interventions to improve NBS disclosures for Krabbe disease

CFIR 2.0 Domain	Representative Quotation
Inner Setting	
Handouts for primary care clinicians with key facts about Krabbe disease	“I would say like a-a fact sheet for pediatricians, like, “Here are some highlights of things.” You know, it’s basically just like not a script necessarily, but like, “Here are the-the main facts that you need to know.” Like, what are the likely next steps that the family is gonna experience? Um, what are the bullet points that they need to know? And also, I think, for us, then following the kid going forward, what are the things that we need to know? You know, what are some things that maybe the chart should be flagged for that we should be especially aware of?” -Participant #18 “If we had any kind of resource—at least with the newborn screens—of bullet points, like, ‘If this is positive, do this. Refer to these people within this timeframe,’ common questions that would probably come up from parents on the initial visits, like, ‘Is this lifelong? Is this curable? Are there medicines for this?‘” - Participant #1
Sample scripts for disclosing NBS results about Krabbe disease	“For our patients with sickle cell trait, the hematologist oncologists have provided us with sort of their spiel that they give to families and things that the families need to know in terms of what to look out for, things to that effect. And so I think having that available on hand … would hopefully improve the comfort level of the provider in sharing those results.” - Participant #10
Algorithms for primary care in managing Krabbe disease	“I know < academic children’s hospital>’s put together before different workflows for common conditions. I don’t know if that—if this is anything that’s available out there for—like, ‘If you have this positive screen, next steps would be X, Y, and Z.‘” - Participant #1 “Um, yeah, the only other thing I can think of is, like, you know, if there was some, like, succinct guideline, like published, written material. So, I guess it would be, you know, A, talking to someone and then, B, having a, like, I guess guideline of, um, that that’s a written guideline of kind of like written for the provider, I should say, that’s—-is positive. You know, these are immediate next steps. These are the symptoms that if the patient has, they should go to the ER. these are the symptoms that if the patient has, they should call us. We are, I think, as a medical community, we’re very used to and comfortable with reading guidelines like that. And they’re extraordinarily helpful. We’re busy. The parents are busy, and, you know, I think having something that would be, you know, a short but detailed enough guideline of-of these are the-the-the basics like, um, could-could be helpful for-for not only Krabbe but a lot of the other, um, metabolic syndromes as well.” - Participant #25 “A little step-wise of if-if somebody screens positive, what the next steps should be from our end in terms of any testing or referrals that we should place.” -Participant #8
Social work support	“[It would be helpful to have] parental supports, meaning like whether that be like some sort of social work” -Participant #25 “Having a case worker that would help facilitate their appointments will be great—uh, you know, to, uh, schedule their genetic, uh, appointment say, “Hey, we’re-we’re calling you.” After the pediatrician has shared the information and they are aware of it and got the knowledge of the diagnosis, then have a case worker say, “I’m here to help you schedule all your next appointments, uh, provide, uh—help you provide transportation if you need it to those appointments, so we can make sure your baby is well taken care of.” - Participant #6
Educational resources for families	“I think making the resources handouts [would be] very accessible to the patients. Even a frequently asked question flyer, just a general overview so that when they leave the visit and things have settled, I’m sure they still have lingering questions or they kind of forget maybe what all they heard. So I would just say making it very accessible to the patient. Whether it was through like the app, like the [patient portal] or, you know, if we emailed them following the visit with the resources in a link.” - Participant #11 “ I think maybe a more simple, basic handout or print material would be helpful if it just kinda breaks down, like, what is the disease and what are the next steps, um, that you could discuss with the family, and then they could also take it home or a—maybe an online resource that they could look up.” - Participant #8 “Because a lot of times then [the families] go home and have lots of questions, even like a frequently asked question pamphlet, for them that we could hand to them and say, this may answer some other questions for you until we can get you to the specialist. Because I feel like, maybe they don’t see the specialist for five or seven days, but as a parent, that’s probably the longest five to seven days of my life. You know, and so if I can—and if I can have something like in writing on paper and I’d rather have something that I can print out and hand to them rather than email to them because they now have a newborn. This is their second kid, they have a newborn, they don’t really have time to, you know, to get—sometimes they only have time to go to the bathroom let alone get on the computer— but I feel like if I can give them a paper, they’re more apt to read a paper at this point in time.” - #23
Outer Setting	
Integration of education with NBS result notification	“I think in general, for pediatricians, if something comes back positive, let’s say a patient screened positive for Krabbe disease is not only notifying the pediatrician by sending some medical information and with the newborn screen about Krabbe disease to that pediatrician. …. Like, information sent about that particular positive screen is sent with the notification about the positive screen.“- Participant #6 “Depending on your community, if there was something that came with the newborn screen to the PCP that said, you know, this screened positive for Krabbe A disease; based on your location, please contact, uh, blank if you need further information on this disease, and-and then how to disclose to the family or next steps for the family.” - Participant #24 “One thing I’ve always thought would be amazing is if there was something that came with the newborn screen that if you have a positive, it’d show you this is what this disease is. You could say that, uh—risk to siblings. This—these are what the next steps should be, right? That—so, instead of needing to actually, like, go look all this stuff up, figure it out and hope that the resources that I found were correct, that it’s just there.” - Participant #14
Improved care coordination with specialists	“I think coordination. I think having us and the specialists kind of coordinating, um, is really important, and so that they don’t get different information from different people. That tends to be a big point of frustration for families in any case.” -Participant #18 “It would be easier just having somebody who has more knowledge - being able to reach out to somebody. I don’t know exactly who that person would be, per se, if it would be just whoever happens to be on call or if there was a point person for a positive newborn screen for either that particular illness or that kinda category of illness. So, I think getting, um, a little bit of guidance from someone who might know more would certainly make it easier.” - Participant #25
Better education and expectation-setting about the NBS in the birth hospital	“ So coming from the newborn nursery, or if the kids are in the NICU, or from the hospital, setting those expectations up for the family. Then, of course, if the hospital’s able to communicate that with us in the outpatient setting, that would be ideal, but somewhere in the notes so that we recognize that they had this conversation with them. They’re aware. So we’re just kinda picking up from there.” - Participant #21

## Barriers

### Individual (clinician-level)

#### Limited knowledge of Krabbe disease

Clinicians uniformly expressed that limited knowledge of Krabbe disease, both their own and that of their colleagues, represents a major barrier to disclosure of positive NBS results. They expressed that, given the rarity of Krabbe disease, pediatricians do not often care for patients with this condition and therefore have limited knowledge of information families might want at time of result disclosure, such as about clinical symptoms, next steps in management, prognosis, current treatment options, and next steps in management.

Participant #25 described this key barrier: “I think it would just be…discomfort with that particular diagnosis… lack of knowledge, lack of experience or exposure… where it is truly one of those things where, I would gander that most outpatient [pediatricians] have not had a patient directly with that, or if they have, it’s been very few.”

This limited knowledge extends to uncertainty around which specialists to contact or refer families for further management. As Participant #7 said, “Sometimes even things like call your local specialist, like—who do I call? Do I call genetics? Are they the right people? Like, who’s the right person to call with it? Like something they’re very clear, oh, this is thyroid, this is an endocrine issue, this is a pulmonary issue. But some of those metabolic genetic stuff, I don’t—yeah, you might call genetics, but it feels a little less clear to me.”

#### Limited interest in learning about rare diseases

Some clinicians expressed a lack of motivation in learning about rare diseases such as Krabbe disease given that they do not encounter rare diseases regularly in their clinical practice. As Participant #2 said, “I think that you would probably not get much turnout for a conference solely on Krabbe disease because it is not that common and people are not gonna feel like it’s worth their time.”

#### Limited comfort in disclosing serious news

Participants discussed the challenges of disclosing serious news to a family such as a NBS result positive for Krabbe disease. As Participant #5 said, “Honestly, I just think the whole thing would be challenging, ‘cause–it’s a big deal-the diagnosis is. So, I think that would be… just if someone’s delivering bad news about your, perfect little newborn. So I think that’d be very stressful and terrible, and I would be, like, really nervous for sure before the conversation.” Similarly, Participant #11 said, “Delivering news like this is always something I feel like providers are uncomfortable doing.”

### Inner setting (clinic-level)

#### Limited time in the clinical schedule

Participants observed that patient volume and limited time in the clinical workday could complicate NBS result disclosure by limiting time for preparation before the visit and time for conversations during the visit, which participants envisioned could be extensive. As Participant #8 said, “I think… the time constraints of our day…. being particularly busy and… making sure that you have enough time to be able to sit down and give your undivided attention to the parents.” Participant #13 similarly expressed, “In the back of your brain, t’s hard to give bad news in a 15-minute window”.

#### Lack of a pre-existing relationship between the clinician and the family

Participants commented that they are sometimes seeing families for the first time at the point when NBS results are disclosed. Therefore, the lack of a pre-existing relationship and rapport with the family could increase the challenge of disclosing serious news. As Participant #18 said, “Like, a lot of times, families don’t see the same pediatrician every time. I would hope that [this result would be disclosed by] someone [who] had a relationship with them.” While many participants commented on the challenges of no existing relationship with a family, one participant (Participant #12) commented that knowing a family well can also pose challenges: “The flip side is knowing the family so well. If they’re emotional it might be a little more emotional for the provider as well.”

#### Limited family knowledge of Krabbe disease

Providers’ poor knowledge of Krabbe disease is compounded by limited general public awareness of Krabbe disease, which providers argued makes the NBS disclosure process more challenging. As Participant #9 commented, “[Krabbe disease is] not something that’s ever gonna become common knowledge for families.” Participant #4 similarly stated, “Some conditions, like, say, thyroid or even sickle cell… there’s some…level of public health literacy… I don’t think there’s any public health literacy about Krabbe disease. Based on the nature of the condition, I’d be worried it’d be hard to explain to family what is it, just ‘cause they wouldn’t necessarily have, like, a background to build off. I think that would be challenging.”

#### Difficulties connecting with families

Some clinicians mentioned inability to engage families either by telephone or in follow-up appointments to disclose and discuss results as a leading challenge. As Participant #23 said, “I think probably the biggest barrier… you know, we get the newborn screening back ‘cause they named us as pediatrician, but they’ve never followed up with us or we can’t get in touch with ‘em.” These challenges may at times be related to social determinants of health: “It falls through the cracks…for many of my families, given these tremendous social determinants of health challenges that we face out here” (Participant #17).

### Outer setting (health system-, regional-, or national-level)

#### Release of results to families before clinicians can disclose the results first exacerbates family distress and complicates subsequent interactions

Participants discussed that they prefer initially disclosing results to families themselves rather than families learning the results first independently. Participants commented that families sometimes receive the results first due to direct communications from the state health department or release of the results immediately to the patient portal as mandated by the 21st Century Cures Act. Participant #24 expressed, “One of the last things that parents want to have happen is for them to get a result and not know what it means and not even have heard from their healthcare provider yet. And it’s one of the downsides of all the open access that we’ve given to families with their own medical information. It’s good for many reasons, but then, in certain circumstances, it’s not. It causes a lot of anxiety for families that could probably be avoided if they were able to hear about it from their healthcare provider first.”

Compounding this problem is potentially distressing information online about Krabbe disease that families could read in advance of speaking with their healthcare provider. Participant #16 explained, “Most people probably Google it, so then they might be worried about it but more concerned as they are from hearing it from me.”

#### Inefficient transmission of NBS results to clinicians

Participants expressed frustration with inefficient or delayed transmission of NBS results to clinicians which could delay disclosure of actionable findings. Participant #10 explained, “There needs to be better communication about the newborn results in general. Because what’s happening—newborn screens are done at the hospital where the infant is born. But then the child is being seen at a pediatric practice by someone who did not see that child, and so the results maybe going to the wrong practice.”

#### Limited access to specialists

Participants observed difficulty accessing specialists for clinician-to-clinician consultation in advance of sharing NBS results. Participant #20 discussed the challenges of a “pediatrician who is in a more rural area or more remote area where maybe it’s harder to get a hold of an expert…on the phone so that they can prepare themselves a little bit more for the conversation or what the next steps might be and stuff like that.” Participant #7 said, “Getting a hold of the specialists every once in a while sometimes is a challenge and it is an issue and you’re trying to page the right person and you can’t get the right person or you’re not getting a call back.”

Clinicians also commented that there are challenges for families in accessing specialty care for follow-up. These include limited appointment availability or significant travel distances due to concentration of specialists in metropolitan areas. As Participant #8 said, “We’re not a big city in terms of referrals and things…we may be limited on what we’re able to do here, and so trying to tell parents if they need to travel or go elsewhere can be difficult for them financially and-and transportation-wise.”

## Facilitators

### Individual (clinician-level)

#### Prior training and experience in communicating serious news

When asked what would facilitate pediatricians’ ability to share NBS results for Krabbe disease, several clinicians commented that they have received prior high-quality training, either in medical school or Graduate Medical Education, in communicating serious or bad news, which they then would be able to utilize in disclosing NBS results for Krabbe disease. As participant #9 said, “the < local teaching hospital> does a good job of the communications course–and sharing difficult news with families.” Participants also expressed that they would be able to draw upon their experiences in sharing other forms of serious news or difficult diagnoses with patients and families to communicate about Krabbe disease.

#### Sense of responsibility to patients and families

Participants also observed that they have a strong sense of professional responsibility to their patients and families, necessitating that they be responsible for the NBS result disclosure process: “Well, as the primary care pediatrician, if that child is in my care, I will always be sure to present that information to the family. And I will take ownership of it as the primary care pediatrician” (Participant #17).

#### Intellectual curiosity and motivation to learn

While several participants had expressed limited interest and motivation in learning about rare diseases, others expressed that, as healthcare professionals, they feel eagerness and professional responsibility to learn about different medical conditions including Krabbe disease. As Participant #17 said, " I’m always down to learn. I learn about everything and anything ’cause you never know when, I’ll have the sort of surprising situation pop up.”

### Inner setting (clinic-level)

#### Protected time for continuing medical education

Several participants observed that they have protected time built into their clinical schedules for Continuing Medical Education activities, including some that are provided as part of their health system. Participant #22 said: “I really do enjoy the [monthly webinars from the local academic children’s hospital that are] typically over a lunchtime, and I try and sign up for that. I feel like that’s a really helpful way to kind of meet us where we’re at.”

### Outer setting (health system-, regional-, or national-level)

#### Accessibility of specialists

While many participants identified inaccessibility of specialists as a barrier, others cited the accessibility and availability of specialists for consultation as a strength of their health systems. As Participant #18 said, “I’m grateful I could call over there [to the academic children’s hospital] and someone would help me… I love collaborating with the specialists over there.”

#### Trustworthy online medical resources

Many participants expressed that there are helpful general medical resources for clinicians online that they regularly read and that would be helpful in disclosing information about an unfamiliar condition: “I think having the resources. It’s really a big thing. As long as [the clinicians] have the resources, and they have access to information like UpToDate” (Participant #6).

## Ideas for interventions

### Inner setting (clinic-level)

#### Handouts for primary care clinicians with key facts about Krabbe disease

Many participants wanted brief fact sheets with key information for primary care clinicians about Krabbe disease and the NBS to help facilitate results disclosure. Participant #18 succinctly stated, “A fact sheet would be really helpful.” Only two participants were aware of existing resources of this type, including ACT sheets and algorithms from the American College of Medical Genetics and Genomics (ACMG).

#### Sample scripts for disclosing NBS results about Krabbe disease

Participants wanted information about “how to talk about [Krabbe disease] with the family” (Participant #20). Many thought it would be helpful to have “some common phrases or verbiage… especially the specialist might use that could help… spark that discussion or guide that discussion” (Participant #19).

#### Algorithms for primary care in managing Krabbe disease

Participants wanted information–potentially in the form of a flow diagram or algorithm– for what their short-term and long-term steps should be in caring for a child with Krabbe disease. Participant #20 said they wanted “potentially an algorithm of, if this is positive or negative, what then those immediate next steps would be, what those long-term next steps might be. Having a decision tree algorithm to understand what the next steps can be right away for someone who’s maybe a non-expert like myself is often helpful.”

#### Social work support

Participants expressed that it would be helpful to have a social worker or case worker in their clinic who could provide additional support and resources for families who have received NBS results for Krabbe disease or other serious conditions. They stated that such a social worker or case worker could provide both moral support and resources to address logistical challenges such as transportation barriers to specialty appointments.

#### Educational resources for families

Many participants said that they would like accurate, accessible, easily available resources to share with families about Krabbe disease and the NBS, whether print or online materials. As Participant #25 said, “And then, it feels a little impersonal to hand somebody a pamphlet with information, but I think oftentimes, when people are receiving bad news, they may not hear everything that’s being said. So, just reference material, reading material for them to go to when they’re ready.”

### Outer setting (health system-, regional-, or national-level)

#### Integration of education with NBS result notification

Many participants indicated that they would appreciate if the state-based system for notification of NBS results in Pennsylvania included information about the relevant condition (such as Krabbe disease) with a positive result. As Participant #12 said, “When we get the information either from the state about a positive screening, [it would be helpful] if there could be supplemental education with that–like recommended supplemental education.” Similarly, participant #4 said, “Again, in my perfect world, there would be a magical little link [on] the newborn screen that would just connect you to—the information you need. Like, you see it says abnormal. Click here for what to do next. Like, I would love a flow diagram or an algorithm or a review that just landed in your lap with the result.”

#### Improved care coordination with specialists

Participants expressed the desire for better care coordination with specialists though they had a variety of ideas for how improvement might be accomplished. Ideas included better communication between the specialist and the primary care clinician, coordination of messaging (i.e. what is said to the family, what language and terminology is used) between the specialist and primary care clinician, and ability of the primary care clinician’s office to arrange follow up for the family with the specialist office.

Participant #22 expressed a specific idea for a hotline that pediatrics clinicians could call to speak with a specialist about abnormal NBS results: “But if [the local academic children’s hospital] had a specific number for abnormal newborn metabolic screens, ‘this is who you’re going to call with questions.’ Or if there was someone specific to that that you could call and talk—and get someone directly—you know, I don’t know how feasible that is. But I feel like that would be really helpful in the middle of a busy day, to be able to have that resource.”

Participant #5 also expressed an idea for specialists to be available at the time of NBS results disclosure: “I think, certainly, it could be an option if [specialists] in Krabbe could be part of the initial conversation even. That—I think actually would be really helpful because then, you would have all the information and not just kinda the early information that we would be able to share. So I think that actually would be helpful, either, like, in conjunction with the pediatrician or separate but working together.”

#### Better education and expectation-setting about the NBS in the birth hospital

Participants suggested that there should be better information and counseling for families about the NBS in the birth hospital at the time when the NBS is collected. Participant #10 explained, " I just think that there’s a definite disconnect that happens between the newborn screen being drawn at the hospital versus the pediatrician who’s actually seeing the patient and then getting the results.” Similarly, Participant #1 stated, “If there was any kind of explanation for the families, like, “We’re testing for these things, and this is what those things would be,’ that might make it a little bit easier for parents to, I guess, be prepared for a positive result if it comes up.”

## Discussion

Newborn screening of Krabbe disease raises distinctive ethical challenges, that are directly related to how results are communicated to families. The rarity of the conditions, rapidity of the changing diagnostic and therapeutic landscape, creates an ethically fraught situation for clinicians who must communicate the possibility and sometimes not the certainty of a life limiting diagnosis to families of otherwise health appearing newborns. Clinicians in outpatient general pediatrics in our study articulated many barriers to their involvement in disclosure of NBS results for Krabbe disease, though they also conveyed many potential facilitators as well as ideas for interventions to improve the NBS result disclosure process. Participants identified barriers and facilitators at the individual level as well as the inner setting (i.e. clinical level) and outer setting (i.e. health system, regional, or national context). Ideas for interventions at the inner setting (i.e. clinic level) included brief informational handouts, sample scripts, and algorithms for primary care clinicians; social work support embedded in the clinic to support families; and educational resources for families. Ideas for interventions at the outer setting included integration of education for clinicians with the NBS notification system; better care coordination with specialists; and better education and expectation-setting about the NBS in the birth hospital. Identified barriers, facilitators, and ideas may inform interventions to improve the NBS disclosure process for Krabbe disease in the U.S.

Much of the discussion about NBS for Krabbe disease has focused on whether and how to include Krabbe disease on the NBS [14–18]. The prior published literature has largely not addressed how to ensure that positive screening results for Krabbe disease are effectively communicated to families in a sensitive, informative, and actionable manner, especially while navigating conditions of genuine scientific uncertainty or nuanced diagnostic, therapeutic and prognostic landscapes. Several prior studies about communication of NBS results have indicated that families prefer to receive positive NBS results from clinicians with whom they have an already-established relationship, such as their child’s pediatrician as opposed to the state health department or a specialist’s practice [6–10]. Such studies have also highlighted dissatisfaction with how pediatrician practices have communicated about conditions on the NBS [6–10].

There have been several interventions tested previously in the U.S. and the U.K. to improve NBS-related communication both in and out of primary care settings [19–24]. U.S. interventions have targeted either the individual clinician level (i.e. feedback and coaching on clinician NBS-related communication skills) [20, 21] or inner setting level (i.e. quality improvement-based audit and feedback of use of ACMG ACT sheets in clinics) [19]. One U.K. based intervention focused on improving midwife communication about the NBS program in antenatal care [22]. An additional U.K. based intervention included multiple components spanning multiple levels: changes to the NBS card, standardized laboratory proforma, communication checklists for clinicians, and informational resources for families with a positive screen [23, 24]. These interventions do not yet have published clinical evaluations [22–24].

The range of barriers identified by clinicians in our study -- spanning individual, inner, and outer settings -- suggests that a multi-component/multi-level intervention is likely also needed in the U.S. context. Some components could potentially be derived from existing materials. For instance, primary care clinicians in our study desired succinct fact sheets about conditions, along with management algorithms, which have already been created as the ACMG ACT sheets [13].

However, novel components are likely also needed, particularly at the outer setting level to coordinate care between the primary care clinician and the specialist. The previous interventions in NBS result disclosure were not specific to rare diseases (i.e. these were either intended to improve NBS-related practices overall or were specific to more common NBS findings such as for sickle cell trait or cystic fibrosis) [19–24]. In the context of a rare yet serious neurometabolic disease, general pediatrics clinicians emphasized the importance of coordinating care with specialists. Their ideas included being able to reach out to specialists in advance of discussing results with the family, being able to arrange rapid follow-up for the patients and families, or even potentially disclosing the NBS results in a co-located manner with both the primary care clinician and specialist available. The idea of co-located care holds particular promise as a strategy allowing the presence of both an already trusted, established figure in the primary care pediatrician and an expert able to address families’ information needs.

Additional ongoing research includes interviewing families who received positive NBS results for Krabbe disease for their thoughts on optimization of the result disclosure process. We then subsequently plan to incorporate what we learned from clinician and family interviews to collaboratively co-design (i.e. with families, multi-disciplinary clinicians, and other key stakeholders) an intervention to optimize NBS result disclosure for Krabbe disease and potentially other rare neurometabolic conditions in the U.S. The objective will be to ensure that families can receive timely, effective, accurate, and sensitive communication about NBS results for Krabbe disease with appropriate integration and coordination of primary care and specialty care.

There are several limitations to this study. We focused on interviewing clinicians in Western Pennsylvania, where the investigative team is located, to be able to leverage established relationships with area primary care pediatrics practices and therefore facilitate recruitment. Strengths of our approach include that we recruited a sample that is diverse in practice setting (i.e. academic vs. private practice), provider type (i.e. physician vs. APP), and practice location (i.e. urban vs. rural vs. suburban). As a limiting factor, findings may not be generalizable to other parts of Pennsylvania or other U.S. states that include Krabbe disease on the NBS. In addition, while several clinicians in our sample had previously cared for children with Krabbe disease, none had personally experienced discussing positive NBS results for Krabbe disease with a family, particularly given the rarity of the condition. Finally, we did not in this study elicit family perspectives on NBS results disclosure, though doing so is a future direction.

In conclusion, clinicians in general outpatient pediatrics in Western Pennsylvania identified a variety of barriers to their optimal integration into NBS results disclosure for Krabbe disease, spanning the individual levels, inner setting, and outer setting of the Consolidated Framework for Implementation Research. However, they also identified several facilitators and many interventions to improve this process. Given the range of identified challenges and the associated ethical considerations, a multi-component, multi-level intervention is likely needed for optimized care, to ensure both the clinical and emotional complexity of the communication. Such an intervention could draw upon some existing resources (particularly from the ACMG) though likely requires addition of novel approaches for education and coordination of care between primary care and specialty care.

## Data Availability

De-identified data will be made available to any investigator upon request.
